# Resveratrol Alleviates the KCl Salinity Stress of *Malus hupehensis* Rhed

**DOI:** 10.3389/fpls.2021.650485

**Published:** 2021-05-12

**Authors:** Tingting Li, Yuqi Li, Zhijuan Sun, Xiangli Xi, Guangli Sha, Changqing Ma, Yike Tian, Caihong Wang, Xiaodong Zheng

**Affiliations:** ^1^College of Horticulture, Qingdao Agricultural University, Qingdao, China; ^2^Qingdao Key Laboratory of Genetic Improvement and Breeding in Horticultural Plants, Qingdao, China; ^3^College of Life Science, Qingdao Agricultural University, Qingdao, China; ^4^Qingdao Academy of Agricultural Sciences, Qingdao, China

**Keywords:** resveratrol, KCl stress, *Malus hupehensis*, ion homeostasis, oxidative stress

## Abstract

Applying large amounts of potash fertilizer in apple orchards for high apple quality and yield aggravates KCl stress. As a phytoalexin, resveratrol (Res) participates in plant resistance to biotic stress. However, its role in relation to KCl stress has never been reported. Herein we investigated the role of Res in KCl stress response of *Malus hupehensis* Rehd., a widely used apple rootstock in China which is sensitive to KCl stress. KCl-stressed apple seedlings showed significant wilting phenotype and decline in photosynthetic rate, and the application of 100 μmol Res alleviated KCl stress and maintained photosynthetic capacity. Exogenous Res can strengthen the activities of peroxidase and catalase, thus eliminating reactive oxygen species production induced by KCl stress. Moreover, exogenous Res can decrease the electrolyte leakage by accumulating proline for osmotic balance under KCl stress. Furthermore, exogenous Res application can affect K^+^/Na^+^ homeostasis in cytoplasm by enhancing K^+^ efflux outside the cells, inhibiting Na^+^ efflux and K^+^ absorption, and compartmentalizing K^+^ into vacuoles through regulating the expression of K^+^ and Na^+^ transporter genes. These findings provide a theoretical basis for the application of exogenous Res to relieve the KCl stress of apples.

## Introduction

Soil salinity is one of the most harmful adverse factors affecting crop productivity in irrigated land (Hu et al., 2015; Zorb et al., 2019). Current research shows that more than 125 million hectares of land has already been salinized, and the area is still increasing. Due to severe salinization, available arable land has been greatly reduced (Liu M. M. et al., 2020). NaCl and KCl are soluble salts that seriously damage the growth of plants. Among the different plant responses to NaCl stress, the core process is to reject sodium (Na) and absorb potassium (K) to maintain Na^+^/K^+^ balance in the cytoplasm (Zhu, 2016). As an essential nutrient element in plants, K maintains the activities of various metabolic enzymes in cells (Zhu et al., 1998). However, recent research found that a high K concentration could also induce salt stress and inhibit the growth of plants (Hassini et al., 2017). Apple (*Malus domestica* Borkh.) is one of the most productive and economically valuable horticultural crops worldwide. Proper soil conditions and nutrient balance are the key factors to guarantee yield and quality (An et al., 2018). However, potash fertilizer consumption has reached 30 million tons per year worldwide and about 5.6 million tons per year in China (Huang et al., 2019; Wu X. W. et al., 2019). Applying large amounts of potash fertilizer for high apple yield and quality has resulted in serious KCl stress (Zheng et al., 2021). This phenomenon damages the soil structure and limits the sustainable development of apple orchards (Jia et al., 2020). Most studies on salt stress mainly focused on the damage of NaCl stress; however, the molecular mechanism underlying the apple’s response to KCl stress remains unclear.

Salt stress includes osmotic stress and ion toxicity, the two primary reactions inducing the accumulation of reactive oxygen species (ROS) and indirectly leading to oxidative damage in plants. The presence of various stresses inhibits plant growth and energy metabolism, which results in premature aging and even death (Guo et al., 2009; Guo H.Y. et al., 2017). When plants experience KCl stress, excessive K^+^ is absorbed into the cytoplasm through an electrochemical potential gradient due to the large amount of K^+^ in the external environment; this phenomenon breaks the original balance of cytoplasm Na^+^/K^+^ and generates ion toxicity (Park et al., 2016). For K^+^ balance in the cytoplasm, ion transporters regulate the ion balance (Li et al., 2006). The Stellar K^+^-Outward Rectifier (SKOR) is located in the plasma membrane to transport K^+^ from the cytoplasm to outside the cell (Sun et al., 2019). For K^+^ absorption, inward channels KAT1 and KAT2 mediate the K^+^ uptake into the cell. KAT1 and HAK5 are high-affinity transporters that regulate sufficient K^+^ uptake for plant growth (Zhao et al., 2016). Except for K^+^ absorption and efflux, voltage-dependent K^+^ channel (TPKs) and vacuolar K^+^/H^+^ antiporters such as NHX1 and NHX2 are present in the tonoplast to facilitate K^+^ influx and efflux in the vacuoles (Zhu et al., 2018). Furthermore, the Na^+^, Ca^2+^, and Fe^2+^ transporters are involved in the balance of K^+^/Na^+^, Ca^2+^, and Fe^2+^ in the cytoplasm (Latz et al., 2013; Yuan et al., 2016; Li et al., 2017). Excessive K^+^ concentration in the soil environment reduces the water potential of the soil, impedes the water uptake of the plants, and leads to osmotic stress (Yang and Guo, 2018). Osmotic stress reduces the stomatal openings through the guard cells of plant leaves, decreases plant photosynthesis, and affects plant growth and development (Meloni et al., 2003; Campos et al., 2012). Plants regulate the osmotic potential by increasing the concentrations of osmolytes such as proline, glycine betaine, soluble sugar, and soluble protein (Yang and Guo, 2018). Osmotic stress and ion toxicity destroy the selective permeability of the membrane, leading to large electrolyte extravasation and excessive ROS accumulation (Demidchik et al., 2014). Excessive ROS concentrations cause lipid peroxidation, protein oxidation, nucleic acid damage, and enzyme inactivation and result in programmed cell death (Affenzeller et al., 2009; Miller et al., 2010). To cope with this oxidative damage, plants develop enzyme, and non-enzyme systems to eliminate ROS (Ahmad et al., 2010). Plant antioxidant enzymes mainly include superoxide dismutase (SOD), peroxidase (POD), and catalase (CAT), and the non-enzyme system mainly involves ascorbic acid, alkaloids, carotenoids, and flavonoids (Tan et al., 2006; Ahmad et al., 2010). Plants can improve the activities of their antioxidant enzymes, accumulate non-enzyme scavengers to alleviate the oxidative damage (Zhan et al., 2019), and induce the expression of ROS-related genes to eliminate ROS. Rice transcription factor OsMADS25 could ameliorate salt tolerance by directly binding to the promoter of glutathione transferase gene *OsGST4* to improve its expression (Xu et al., 2018).

Applying exogenous substances effectively alleviates salt stress (Su et al., 2020). These substances are commonly divided into the following three categories: The first category is the plant growth regulators, such as jasmonate (JA), cytokinin, and abscisic acid (Moons et al., 1997; Zhu, 2016; Wu J. X. et al., 2019). JA has a positive regulatory role in plant resistance to salt stress, and its exogenous application can improve the salt tolerance of *Arabidopsis*, tomatoes, and rice (Garcia-Abellan et al., 2015; Ken-Ichi et al., 2015; Valenzuela et al., 2016). The second category is osmotic adjustment substances, such as proline, glycine betaine, and sugars (Finkelstein and Lynch, 2000; Yang and Lu, 2005). Exogenous glycine betaine application can alleviate NaCl stress by regulating osmotic stress (Yang and Lu, 2005). The third category contains substances that increase the antioxidant capacity of plants, such as NO, silicon, and melatonin (Hu et al., 2017; Zeng et al., 2018; Zhu et al., 2019). Exogenous melatonin can eliminate ROS and enhance NaCl stress resistance in horticultural crops such as grapes, apple, and cucumber (Zheng et al., 2017; Xu et al., 2019). However, most studies focused on NaCl stress; reports on substances that are effective against KCl stress are rare. As a member of the stilbene family of phenolic compounds, resveratrol (Res) has been identified in grapevines, red wine, sorghum bicolor, berries, and peanuts (Kostopoulou et al., 2014). This antimicrobial phytoalexin contributes to plant resistance to biotic stress (Kiselev et al., 2017). Grapevines could metabolize additional Res to protect themselves from *Botrytis cinerea* and *Plasmopara viticola* (Langcake et al., 1979; Kiselev et al., 2009). Gonzalez Ureña et al. (2003) reported that exogenously applying *trans-*resveratrol could improve postharvest resistance in fruits. Res can also help improve the resistance to *Venturia inaequalis* in apples (Heuer, 2003). In addition to acting as a phytoalexin, Res is involved in plant resistance to abiotic stress and plant response to ozone, wounding, or UV light (Grimmig et al., 2003). In citrus seedlings, the external application of Res and α-Toc mediates salt adaptation (Kostopoulou et al., 2014). However, the effect of Res on KCl stress and its molecular mechanism are still unclear, especially in woody plants such as apples.

In this study, we aimed at exploring the effect and its underlying mechanism of Res on KCl stress in apple. Our study applied different concentrations of exogenous Res on *Malus hupehensis* seedlings under KCl stress and analyzed the positive regulation of Res under KCl stress in terms of the photosynthetic system, ionic homeostasis, osmotic balance, and oxidative damage. Additionally, K^+^ and Na^+^ transporter genes and key KCl-responsive genes under Res treatments were also determined. Our results suggest that Res alleviates the KCl salinity stress of *Malus hupehensis* seedlings by regulating K^+^/Na^+^ homeostasis, osmotic adjustment, and ROS scavenging. The results of this study shed light on the new effect of Res and enhance the application of Res in apples under abiotic stress.

## Materials and Methods

### Plant Materials and Growth Conditions

Seeds of *M. hupehensis* after low-temperature vernalization were planted in nutrient soil (65% fertile garden soil, 10% fine sand, 25% burning soil, and 0.4% calcium magnesium phosphate fertilizer) and sand with the ratio of 1:1. When the seedlings developed to four leaves, they were transplanted into a 7 × 7 × 8-cm plastic pot, watered with Hoagland’s nutrient solution every 3 days, and cultivated under a greenhouse environment with the temperature controlled at 25 ± 2°C, humidity of 62 ± 2%, light intensity of 100 μmol⋅m^–2^⋅s^–1^ and photoperiod of 16/8-h light/dark. After 10 days, the seedlings with similar growth status were selected for subsequent KCl and exogenous Res treatment.

### KCl Stress and Exogenous Res Treatment

A total of 360 *M*. *hupehensis* seedlings were randomly divided into five groups, and each group had 72 apple seedlings. Group I was treated with Hoagland’s nutrient solution as control, and groups II–V were irrigated with 50 mM KCl stress (KCl dissolved into Hoagland’s nutrient solution). In addition, groups III–V were sprayed with exogenous Res with a concentration of 10, 100, and 200 μM, respectively; Res (Solarbio, Beijing, China) was dissolved in ethanol at a concentration of 10 mM and stored at −20°C and sprayed every 2 days. The Hoagland’s nutrient solution with/without KCl stress were irrigated every 3 days. After 15 days of treatment, the phenotype was photographed firstly, then the wilting rate, plant height, fresh weight, and dry weight of the apple seedlings were measured. Both the biological and technical duplications of each experiment were repeated three times.

### Determination of Wilting Rate, Plant Height, Fresh Weight, and Dry Weight

After 15 days of KCl stress and exogenous Res treatment, the wilting rates of the apple seedlings in five groups were counted (wilting rate = number of wilting seedlings/total number of seedlings in each group × 100%). Twenty seedlings from each group were randomly selected to measure the plant height. The distance from root collar to the top of the stem was measured as plant height using a ruler. Then, the 20 apple seedlings were washed and dried with filter paper. The fresh weight was detected by analytical balance (Mettler toledo, Zurich, Switzerland). For the measurement of dry weight, the apple seedlings were dehydrated at 105°C for 30 min and then baked at 80°C for continuous 72 h; then, dry weight was measured using analytical balance (Mettler Toledo, Zurich, Switzerland). Both the biological and technical duplications of each experiment were repeated three times.

### Determination of Chlorophyll Content and Photosynthetic Parameters

Twenty apple seedlings from each group were randomly selected to determine the chlorophyll content and basic photosynthetic parameters after KCl stress and exogenous Res treatment for 15 days. Three leaves of each seedlings were measured. Under light condition, the chlorophyll content was measured by SPAD-502 Plus (Konica Minolta, Tokyo, Japan) at 9 am to 11 am. Photosynthesis rate, transpiration rate, and stomatal conductance were measured by CIRAS-3 portable photosynthetic apparatus (PP Systems, Amesbury, United States). Light intensity was set at 800 μmol⋅m^–2^⋅s^–1^ supplied by the built-in light source, humidity was 50%, and temperature was controlled at 23°C. The photosynthetic parameters were all measured between 9 am and 11 am. Both the biological and technical duplications of each experiment were repeated three times.

### Determination of ROS Level and MDA Content

Twenty apple seedlings, after KCl stress and exogenous Res treatment for 15 days, were randomly selected from each group to detect the ROS levels, including O_2_^.–^ and H_2_O_2_. For O_2_^.–^ measurement, the leaves were vacuum-infiltrated with 0.1 mg/ml nitroblue tetrazolium (Sangon Biotech, Shanghai, China) in 25 mM HEPES buffer (pH = 7.8) for 30 min and kept at 23°C in the dark for 2 h. For the detection of H_2_O_2_, the leaves were vacuum-infiltrated with 0.1 mg/ml diaminobenzidine (Sangon Biotech, Shanghai, China) in 50 mM Tris-acetate (pH = 3.8) for 30 min and were kept at 23°C in the dark for 24 h. Then, leaves for either O_2_^.–^ or H_2_O_2_ detection were washed in 80% ethanol every 10 min at 70°C until the leaves lost their green color completely (Zheng et al., 2017). After decoloration, the leaves were photographed. Each experiment was independently repeated three times.

For malondialdehyde (MDA) detection, 0.5 g of fresh leaves from each group after KCl stress and exogenous Res treatment for 15 days were ground in a pre-cooled mortar with 5 ml of extract buffer and then centrifuged at 12,000 rpm for 10 min. MDA content was determined using a plant MDA extraction kit (Grace, Suzhou, China). Both the biological and technical duplications of each experiment were repeated three times.

### Determination of Antioxidant Enzyme Activity

After KCl stress and exogenous Res treatment for 15 days, 0.5 g of fresh leaves from each group was ground in 5 ml of extract buffer to determine the activity of antioxidant enzymes including SOD, POD, and CAT in apple leaves. After centrifugation at 12,000 rpm for 10 min, the activities were determined using SOD, POD, and CAT kits (Grace, Suzhou, China). Both the biological and technical duplications of each experiment were repeated three times.

### Determination of Electrolyte Leakage and Osmolyte Content

Twenty leaves after KCl stress and exogenous Res treatment for 15 days were randomly selected from each group to detect electrolyte leakage. The detection of electrolyte leakage was estimated as described previously (Ahmad et al., 2016). Firstly, the electrical conductivity (ECa) of the 20 leaf disks submerged was measured. Secondly, the leaf disks were put in test tubes and incubated at 55°C for 25 min, and the electrical conductivity (ECb) was measured. Finally, the test tubes were boiled at 100°C for 10 min, and the electrical conductivity (ECc) was determined. Electrolyte leakage was calculated using the following formula: electrolyte leakage (%) = (ECb – ECa)/ECc × 100. Both the biological and technical duplications of each experiment were repeated three times.

After KCl and exogenous Res treatment for 15 days, 0.5 g of fresh leaves from each group was used for the detection of osmolytes including proline, soluble sugar, and soluble protein. The leaves were ground in 5 ml of pre-cooled extracted buffer, and centrifugation (12,000 rpm) was carried out at 4°C for 10 min. The supernatants were used for proline, soluble sugar, and soluble protein content assays using the proline, soluble sugar, and soluble protein kits (Grace, Suzhou, China). Both the biological and technical duplications of each experiment were repeated three times.

### Quantification of Mineral Elements

A total of 3 g of apple seedlings were collected after 15 days of KCl stress and Res treatment and then cleaned with deionized water. The plants were dehydrated at 105°C for 30 min and then baked at 80°C for 72 h. Afterward, 0.5 g of dried seedlings was ground into powder and added with 12 ml of HNO_3_ and HClO_4_ (ratio of 5:1). After digestion, the solution was diluted with deionized water to 25 ml for the detection of mineral elements. The macronutrient (K, Ca, Na, Mg, and P) and micronutrient (Fe, Mn, Zn, and Cu) contents were determined by inductively coupled plasma-optical emission spectrometry (PerkinElmer, Waltham, United States). Both the biological and technical duplications of each experiment were repeated three times.

### RNA Extraction and Quantitative Real-Time PCR Analysis

After 15 days of KCl and Res treatments, the total RNA of different groups was extracted using the RNAprep pure Plant Plus Kit (Tiangen, Beijing, China). Inverse transcription and qPCR assay were conducted as described by Zheng et al. (2020). KCl-responsive genes in apple were screened from RNA-seq results (NCBI number PRJNA588566), and apple actin (accession number: MDP0000774288) was used as the internal reference. The primers used for qPCR were designed by Primer 5 software and are shown in Supplementary Table 1. Both the biological and technical duplications of each experiment were repeated three times.

### Experimental Design and Statistical Analysis

All experiments were repeated thrice according to a completely randomized design. The data were analyzed by ANOVA followed by Fisher’s least significant difference or Student’s *t*-test analysis. Statistically significant differences were indicated by *P* < 0.05. Statistical computations were conducted using SPSS (IBM, Armonk, NY, United States).

## Results

### Effects of Exogenous Res on the Growth of Apple Seedlings Under KCl Stress

**FIGURE 1:**
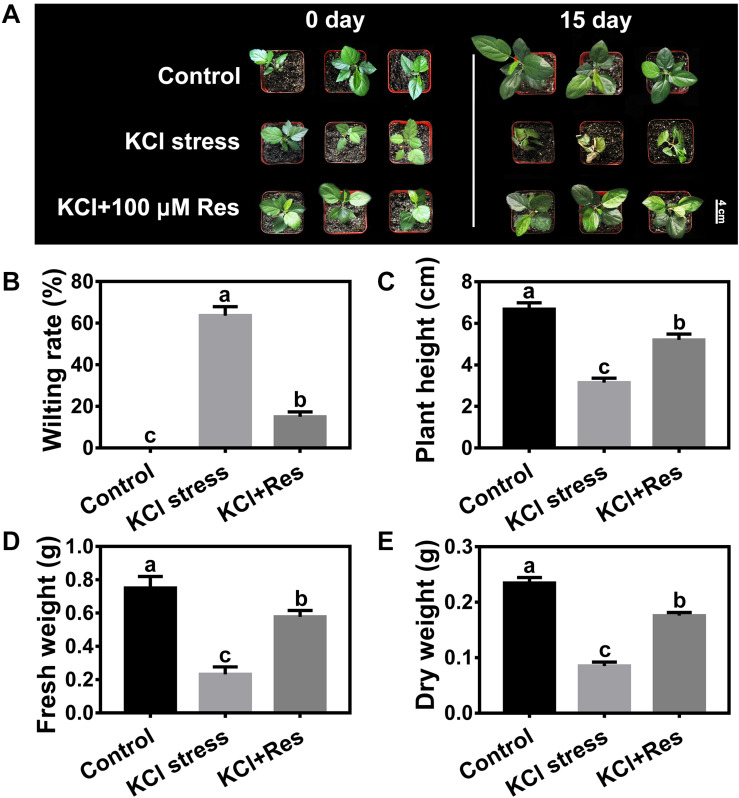
Phenotypes of *Malus hupehensis* seedlings treated with 50 mM KCl stress and exogenous 100 μmol Res on day 0 and day 15 **(A)**. Effect of Res on wilting rate **(B)**, plant height **(C)**, fresh weight **(D)**, and dry weight **(E)** of apple seedlings after KCl stress for 15 days. The bar **(A)** represents 4.0 cm. The data represent the mean ± SD of biological replicates. Different lowercase letters indicate significant differences according to Fisher’s least significant difference (*P* < 0.05).

As shown in Supplementary Figure 1, the seedlings were wilted and seriously damaged by 50 mM KCl stress. When different Res concentrations were applied to the KCl-stressed apple seedlings, the leaves were still kept green, and the wilting rates were significantly reduced (Supplementary Figure 1A). However, different degrees of protection were observed when spraying varying Res concentrations under KCl stress. When low (10 μM) and high concentrations (200 μM) were used, the wilting rates of the apple seedlings were significantly decreased from 68.9 to 38.9 and 41.1%, respectively (Supplementary Figure 1B), and the fresh weights were significantly increased to 104 and 39.5%, respectively (Supplementary Figure 1C). When 100 μM exogenous Res was applied, the wilting rate was significantly decreased to as low as 15.0% compared with that of group II (Figure 1B). In addition, the plant height, fresh weight, and dry weight were all remarkably increased in the seedling sprayed with 100 μM Res compared with that without exogenous Res under KCl stress for 15 days (Figure 1). The plant height decreased from 6.7 to 3.2 cm under KCl stress but recovered to 5.2 cm after 100 μM Res application (Figure 1C). The fresh and dry weights also significantly increased to 148 and 107%, respectively, compared with those of group II under KCl stress for 15 days (Figures 1D,E). These results indicated that exogenous Res could protect the apple seedlings from KCl stress. The treatment of 100 μM exogenous Res exhibited the best phenotype and was therefore selected for further research.

### Effects of Exogenous Res on Chlorophyll Content and Photosynthetic Parameters Under KCl Stress

**FIGURE 2:**
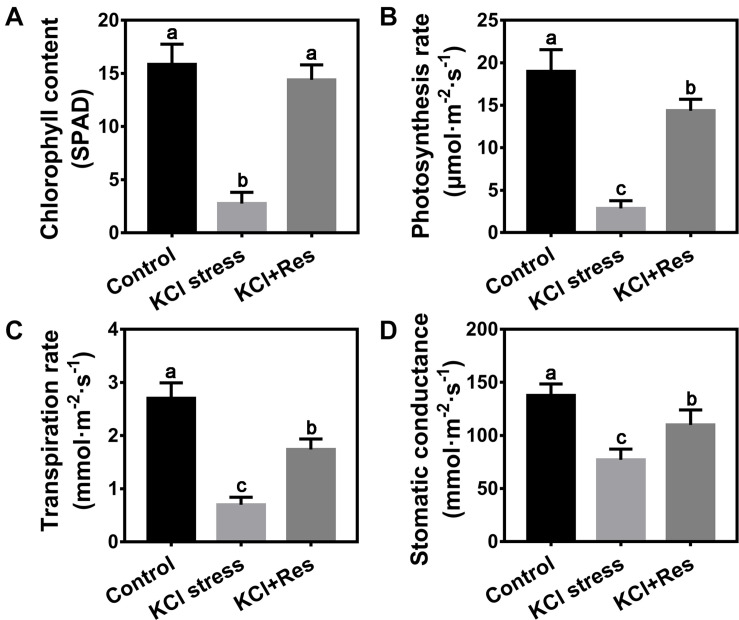
Effects of Res treatment on chlorophyll content **(A)**, photosynthesis rate **(B)**, transpiration rate **(C)**, and stomatic conductance **(D)** of apple seedlings under KCl stress. The data represent the mean ± SD of biological replicates. Different lowercase letters indicate significant differences according to Fisher’s least significant difference (*P* < 0.05).

In consideration of the wilting phenotype on the leaves of apple seedlings under KCl stress, the chlorophyll content and photosynthetic parameters were measured. The chlorophyll content was significantly reduced in the plants after 15 days of KCl stress (2.75 SPAD) and was only one-fifth that of the control group (15.85 SPAD). When exogenous Res was sprayed, the chlorophyll content of apple seedlings under KCl stress significantly recovered to as high as 14.4 SPAD, with no significant difference from that of the control group (Figure 2A). A similar variation tendency was observed for the photosynthetic parameters including photosynthesis rate, transpiration rate, and stomatic conductance under KCl stress and exogenous Res treatment. All values were significantly inhibited under KCl stress but increased by exogenous Res application (Figures 2B–D), especially the photosynthesis rate. Under KCl stress, the photosynthesis rate decreased significantly from 19 to 2.85 μmol⋅m^–2^⋅s^–1^ but recovered to 14.35 μmol⋅m^–2^⋅s^–1^ when exogenous Res was applied (Figure 2B). These results indicated that exogenous Res could protect the chlorophyll level and photosynthetic system against KCl stress.

### Effects of Exogenous Res on the Oxidative Damage and Antioxidant Enzyme Activity of Apple Seedlings Under KCl Stress

**FIGURE 3:**
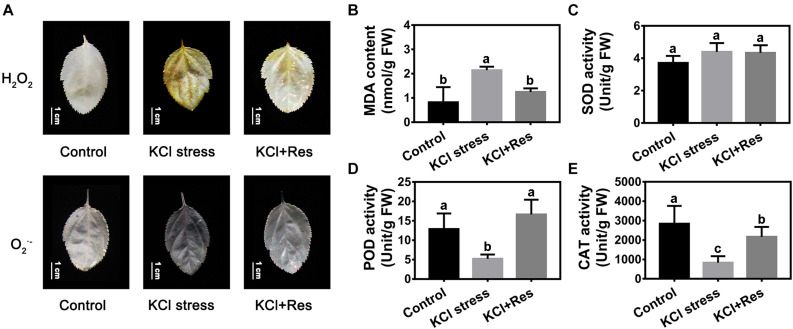
Effects of Res treatment on H_2_O_2_, O_2_^–^ **(A)**, and malondialdehyde (MDA) content **(B)**, superoxide dismutase activity **(C)**, peroxidase activity **(D)**, and catalase activity **(E)** under KCl stress. The bar **(A)** represents 1.0 cm. The data represent the mean ± SD of biological replicates. Different lowercase letters indicate significant differences according to Fisher’s least significant difference (*P* < 0.05).

The O_2_^.–^ and H_2_O_2_ staining results revealed that the leaves of apple seedlings were seriously damaged by KCl stress for 15 days. When exogenous Res was applied, the O_2_^.–^ and H_2_O_2_ levels were significantly decreased (Figure 3A). The variation tendency of the MDA content was similar to that of O_2_^.–^ and H_2_O_2_ under KCl and Res treatment. The MDA content under KCl stress (2.17 nmol/g) was more than twice that of the control group (0.83 nmol/g) but was significantly decreased to as low as 1.28 nmol/g after exogenous Res was applied (Figure 3B).

Superoxide dismutase, POD, and CAT activities were also detected. As shown in Figure 3C, SOD activity was not significantly changed under KCl stress and exogenous Res treatment. Different from that of SOD, the POD and CAT activities under KCl stress were significantly decreased from 13.2 and 2,813 to 5.09 and 905 U/g, respectively. However, when exogenous Res was applied, the POD activity recovered to 16.3 U/g, which was as high as that under normal conditions (Figure 3D), and the CAT activity significantly increased to 2,177 U/g (Figure 3E).

### Effects of Exogenous Res on the Electrolyte Leakage and Osmolytes of Apple Seedlings Under KCl Stress

**FIGURE 4:**
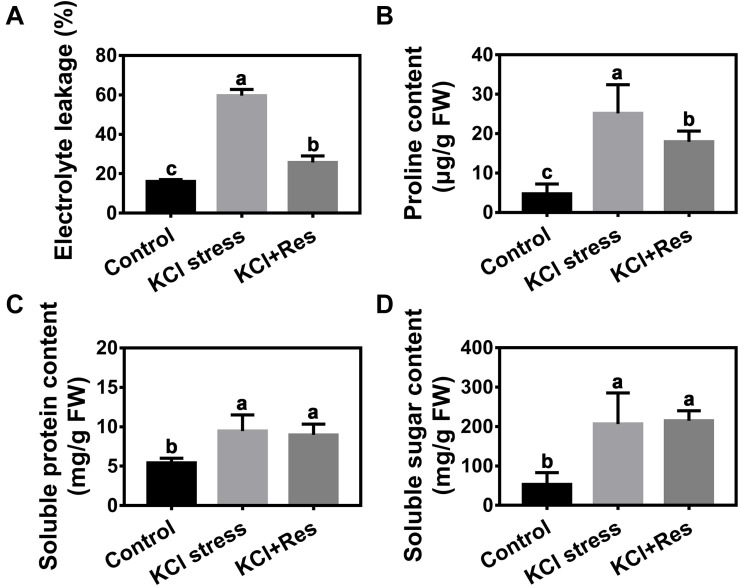
Effects of Res treatment on electrolyte leakage **(A)**, proline content **(B)**, soluble protein content **(C)**, and soluble sugar content **(D)** under KCl stress. The data represent the mean ± SD of biological replicates. Different lowercase letters indicate significant differences according to Fisher’s least significant difference (*P* < 0.05).

Electrolyte leakage was detected after KCl stress and exogenous Res treatment for 15 days. After KCl stress, the electrolyte leakage increased significantly from 16.07 to 59.6% but decreased to as low as 28.9% when exogenous Res was applied (Figure 4A).

Osmolyte content under KCl stress and exogenous Res treatment was also detected. As shown in Figure 4, the proline, soluble sugar, and soluble protein contents were all significantly increased by KCl stress. However, when exogenous Res was applied, the proline content was significantly decreased from 25.2 to 17.3 μg/g, and those of soluble sugar and soluble protein content had no significant changes.

### Effects of Exogenous Res on the Mineral Elements of Apple Seedlings Under KCl Stress

**FIGURE 5:**
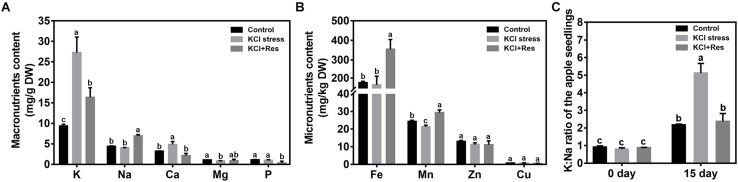
Effects of exogenous Res treatment on macronutrient content **(A)**, micronutrient content **(B)**, and K/Na ratio **(C)** under KCl stress. The data represent the mean ± SD of biological replicates. Different lowercase letters indicate significant differences according to Fisher’s least significant difference (*P* < 0.05).

The mineral elements of apple seedlings were measured after KCl stress and exogenous Res treatment for 15 days. For the macronutrients (Figure 5A), K level was significantly increased from 9.06 to 27.25 mg/g under KCl stress but decreased to 16.34 mg/g when exogenous Res was applied. The variation tendency of Ca was similar to that of K. Different from those of K and Ca, Na content had no significant changes under KCl stress. However, when exogenous Res was applied, Na significantly increased by 56.9%. For the micronutrients (Figure 5B), Mn was reduced by KCl stress but significantly increased from 21.27 to 28.96 mg/kg when exogenous Res was applied. The Fe content had no significant changes under KCl stress but significantly increased by 100.5% when exogenous Res was applied. As an important indicator of plant tolerance to salt stress, K/Na ratio was detected before and after KCl stress and exogenous Res treatment for 15 days. The K/Na ratio of the apple seedlings from the three groups had no significant difference before the treatment. However, after KCl stress and exogenous Res treatment for 15 days, the K/Na ratio was significantly increased to 489% under KCl stress but decreased from 5.3 to 2.4 after exogenous Res treatment (Figure 5C).

### Effects of Exogenous Res on the Expression Levels of KCl-Related Genes in Apple Seedlings Under KCl Stress

**FIGURE 6:**
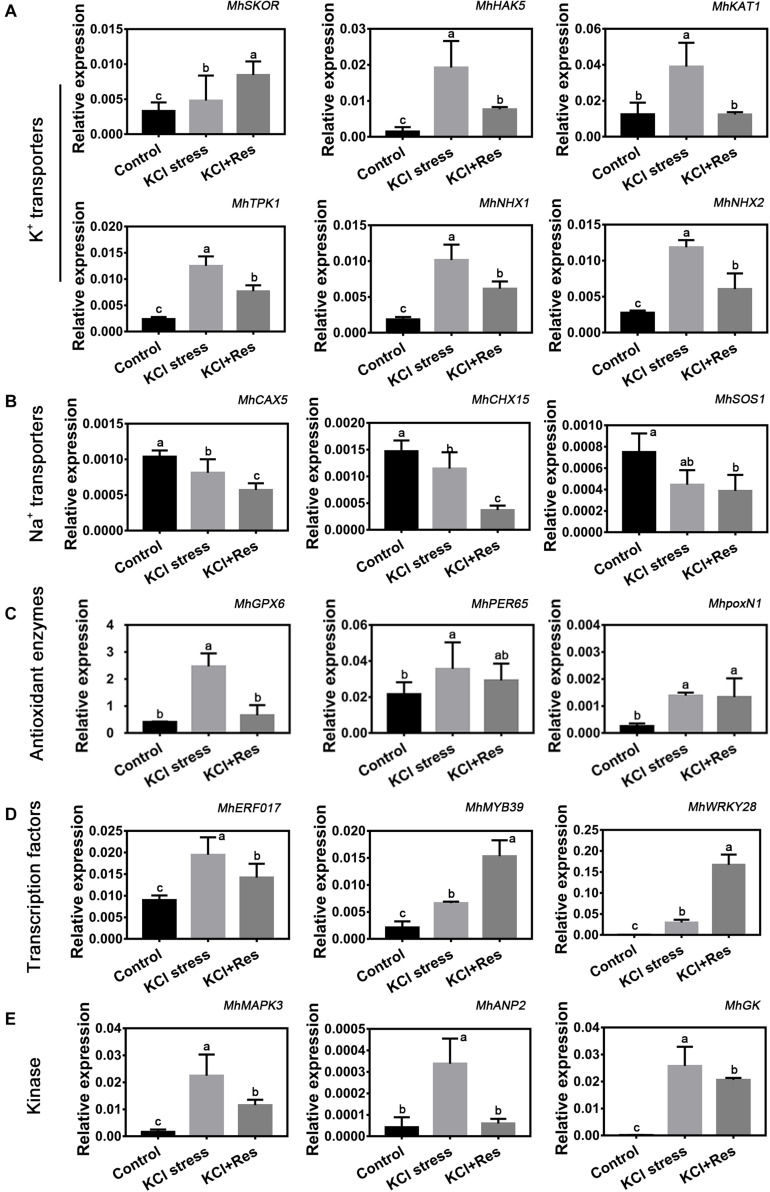
Eighteen candidate genes are divided into K^+^ transporters (*MhSKOR*, *MhHAK5*, *MhAKT1*, *MhTPK1*, *MhNHX1*, and *MhNHX2*) **(A)**, Na^+^ transporters (*MhCAX5*, *MhCHX15*, and *MhSOS1*) **(B)**, antioxidant enzymes (*MhGPX6*, *MhPER65*, and *MhpoxN1*) **(C)**, transcription factors (*MhERF017*, *MhMYB39*, and *MhWRKY28*) **(D)**, and kinase (*MhMAPK3*, *MhANP2*, and *MhGK*) **(E)**. The expression of the 18 candidate genes under KCl stress and exogenous Res treatment for 15 days. The data represent the mean ± SD of biological replicates. Different lowercase letters indicate significant differences according to Fisher’s least significant difference (*P* < 0.05).

As shown in Figure 6, the expression levels of 18 candidate genes, which were screened out from RNA-Seq data under KCl stress, were detected under KCl and exogenous Res treatment. These genes were categorized into five groups. First, the six K^+^ transporter genes including *MhSKOR*, *MhHAK5*, *MhKAT1*, *MhTPK1*, *MhNHX1*, and *MhNHX2* had a significantly increased expression under KCl stress. However, those of *MhHAK5*, *MhKAT1*, *MhTPK1*, *MhNHX1*, and *MhNHX2* were significantly downregulated, whereas that of *MhSKOR* was further upregulated by exogenous Res treatment (Figure 6A). Second, three Na^+^ transporter genes including *MhCAX5*, *MhCHX15*, and *MhSOS1* showed a similar decreasing tendency under KCl stress and exogenous Res treatment (Figure 6B). Third, the expression of antioxidant enzyme genes *MhGPX6*, *MhPER65*, and *MhpoxN1* was significantly induced by KCl stress, and only that of *MhGPX6* was significantly affected by exogenous Res treatment (Figure 6C). Finally, the expression of three selected transcription factors, namely, *MhERF017*, *MhMYB39*, and *MhWRKY28*, and three kinases, namely, *MhMAPK3*, *MhANP2*, and *MhGK*, was also significantly changed under KCl and exogenous Res treatment. This finding indicated their potential important functions under plant response to KCl stress and Res signaling transduction pathway.

## Discussion

Potash is applied as a fertilizer to fulfill the requirement of K, which is an essential nutrient for plant growth and physiology (Hasanuzzaman et al., 2018; Huang et al., 2019). K has a regulatory function in several biochemical and physiological processes, such as protein synthesis, enzyme activation, carbohydrate metabolism, and photosynthesis (Zhu et al., 1998; Huang et al., 2018; Wu X. W. et al., 2019). However, K^+^ at concentrations higher than 50 mM can induce salt stress and disrupt normal plant growth and metabolism (Hassini et al., 2017; Ntatsi et al., 2017). *M. hupehensis* is one of the most popular rootstocks for apple production and cultivation (Su et al., 2020) but unfortunately suffers from serious KCl stress due to the huge amount of potassium fertilizer applied to orchards (Zheng et al., 2021). Introducing exogenous substances such as plant growth regulator, osmotic adjustment substances, and antioxidants effectively alleviates salt stress (Li et al., 2012; Chen et al., 2019; Hamani et al., 2020). As an antimicrobial phytoalexin, Res has an effect on high boron in pepper plants and alleviates NaCl stress in citrus seedlings (Kostopoulou et al., 2014; Sarafi et al., 2017). However, the roles and molecular mechanism underlying Res activity on KCl stress have never been reported. In this study, the role of different Res concentrations was examined in *M. hupehensis* seedlings under KCl stress. The treatment of 100 μmol Res for KCl stress produced the lowest wilting rate and the highest fresh weight and therefore had a better effect than 10 μmol (low concentration) and 200 μmol (high concentration) (Figure 1 and Supplementary Figure 1). Exogenous plant regulators usually affect plant growth and development in a dose-dependent manner. In *Malus baccata* seedlings, 600 μmol for irrigation or 200 μmol for spraying is selected as the best concentration of melatonin to maximize its role under waterlogging stress (Zheng et al., 2017). Our results revealed that 100 μmol Res would be an appropriate concentration to alleviate KCl stress in *M. hupehensis* seedlings. This is consistent with the efficient concentration of Res to NaCl stress in citrus seedlings (Kostopoulou et al., 2014). Therefore, 100 μmol Res would be an appropriate concentration to alleviate salinity stress in woody plants like apple and citrus.

Under KCl stress, the direct injury to plants is called ion toxicity (El-Zawily et al., 2019). Large amounts of K^+^ flow into the cytoplasm and cause an imbalance between the cations. When the apple seedlings were under KCl stress, the K content was sharply induced, and that of Ca was also substantially increased (Figure 5). Ca is an important secondary messenger, and maintaining its concentration in the cytoplasm helps to regulate plant signal transduction pathways under salt stress (Ding et al., 2010). Therefore, increasing the Ca content must be the stress response of the apple seedlings to balance K^+^/Ca^2+^ in the cytoplasm. When exogenous Res was applied, the decreased K content and increased Na, Fe, and Mn indicated that Res could affect the ion transport under KCl stress. Fe and Mn play a key role in plant resistance to oxidative stress (Yuan et al., 2016; Kohli et al., 2019), so the increase of their contents after Res treatment would be the response to oxidative damage caused by KCl stress. In plant responses to salt stress, the core process is the balance of K^+^/Na^+^ in the cytoplasm (Ashraf and Harris, 2004; Hasanuzzaman et al., 2014). K^+^/Na^+^ ratio was significantly induced under KCl stress but decreased to control level when exogenous Res was applied (Figure 5). To investigate the cause of the K^+^/Na^+^ ratio change, the expression of K^+^ and Na^+^ transporter genes responding to KCl stress was analyzed from previous RNA-seq data (NCBI number: PRJNA588566). First, SKOR family, which is located in the plasma membrane, is mainly responsible for K^+^ efflux from the cytoplasm to outside of the cell. HAK and KAT proteins function for K^+^ absorption (Sun et al., 2019). The present study indicated that *MhSKOR* expression was sharply induced by KCl stress and even enhanced by exogenous Res application, and the expression levels of *MhHAK5* and *MhKAT1* were induced by KCl stress but inhibited by exogenous Res application (Figure 6). These results indicated that exogenous Res could enhance K^+^ efflux and inhibit K^+^ influx under KCl stress. Second, for the compartmentalization of K^+^ in cells, the two-pore-channel TPK1 gene encodes the vacuolar K^+^ conductance and plays a role in K^+^ homeostasis (Wang et al., 2018). Vacuolar K^+^/H^+^ antiporters NHX1 and NHX2 are present in the tonoplast to facilitate K^+^ influx and efflux in the vacuoles (Zhu et al., 2018). In this study, the expression levels of *MhTPK1*, *MhNHX1*, and *MhNHX2* were induced by KCl stress and inhibited by exogenous Res treatment but remained higher than the control level (Figure 6). We hypothesized that *MhTPK1*, *MhNHX1*, and *MhNHX2* could function to compartmentalize K^+^ into the vacuoles to balance K^+^ homeostasis in the cytoplasm under KCl stress. When exogenous Res was applied, the expression of these genes was reduced to regulate K^+^ influx and efflux in the vacuoles and ensure K^+^ homeostasis in the cytoplasm and vacuoles. Therefore, exogenous Res could affect K^+^ homeostasis in the cytoplasm by enhancing K^+^ efflux outside the cells, inhibiting K^+^ absorption, and compartmentalizing K^+^ into vacuoles under KCl stress. For Na^+^ transport under KCl stress, Na^+^ balance is mainly the result of passive influx and active efflux (Zhu, 2003). Therefore, the expression of Na^+^/H^+^ and cation/H^+^ antiporter genes was detected. The expression of *MhCHX15* and *MhCAX5*, which expel Na^+^ from cells, was significantly inhibited under KCl and Res treatment (Figure 6). These results indicated that exogenous Res could decrease the expulsion of Na^+^ out of the cells to ensure K^+^/Na^+^ homeostasis in the cytoplasm under KCl stress. In summary, exogenous Res could alleviate KCl stress-induced ion toxicity by regulating the transcription of K^+^, Na^+^, and Ca^2+^ transporters and maintaining the homeostasis of K^+^/Na^+^, K^+^/Ca^2+^, K^+^/Fe^2+^, and K^+^/Mn^2+^ in the cytoplasm.

Osmotic stress is another direct injury to plants caused by KCl stress (Guo et al., 2009). The results indicated that electrolyte leakage was significantly induced by KCl stress but inhibited by exogenous Res treatment. This result was in agreement with a previous study which stated that Res could protect plants from osmotic stress (Fernández-Mar et al., 2012). To investigate the function mechanism of Res on osmotic stress, we detected the proline, soluble sugar, and soluble protein contents under KCl and Res treatment. Our results indicated that exogenous Res could affect the proline content but not the soluble sugar and soluble protein contents under KCl stress (Figure 4). Accumulation of osmotic substances, such as proline, soluble sugar, and soluble protein, is a common defense mechanism of plants subjected to salinity (Munns and Tester, 2008). In citrus seedlings, exogenous Res could increase carbohydrates and proline concentration after NaCl treatment (Kostopoulou et al., 2014). Therefore, exogenous Res could protect plants from osmotic stress through affecting the accumulation of proline.

Oxidative damage is the subsequent injury caused by osmotic stress and ion toxicity (Guo et al., 2009). It is believed that some of the beneficial actions of Res are due to its extremely efficient free radical scavenger activity, thus preventing the peroxidative damage of cellular structures (Stojanovic et al., 2001). In this study, the O_2_^.–^, H_2_O_2_, and MDA contents were significantly higher under KCl stress, and exogenous Res could eliminate O_2_^.–^ and H_2_O_2_ and decrease the MDA content under KCl stress in apple seedlings (Figure 3). This was consistent with the fact that Res was reported to function in scavenging ROS and alleviating oxidative damage in cell systems (Fernández-Mar et al., 2012). SOD, POD, and CAT are the three main antioxidant enzymes in enzymatic antioxidant systems (Tan et al., 2012; Abdelaal et al., 2018). The POD and CAT activities were inhibited by KCl stress, and the SOD activity had no change (Figure 3). This finding was consistent with that under NaCl stress (Kostopoulou et al., 2014). Although these enzymes are antioxidants, their activities could be inhibited by serious salt stress. When exogenous Res was applied, the POD and CAT activities were significantly increased, and the SOD activity remained unchanged (Figure 3). SOD is the major element in ROS scavenging (Gupta et al., 2019). Under NaCl stress and Res treatment, the SOD activity was only slightly induced, leading to low H_2_O_2_ production (Hasanuzzaman et al., 2014). However, the SOD activity had no change under KCl and Res treatment. This finding explains the difference of SOD function under KCl and NaCl stresses. POD and CAT activities showed a similar variation tendency under KCl and Res treatment, indicating their important role in ROS scavenging by Res under KCl stress. Furthermore, the expression levels of three ROS-related genes (*MhGPX6*, *MhPER65*, and *MhpoxN1*) screened out from RNA-seq data under KCl stress were also detected under KCl and Res treatment. The results showed that the expression levels of peroxidase gene *MdPER65* and peroxidase N1 gene *MhpoxN1* were significantly induced by KCl stress but showed no change under Res treatment. However, *MhGPX6*, the glutathione peroxidase gene, was significantly induced by KCl stress but inhibited by exogenous Res treatment (Figure 6). Thus, exogenous Res could alleviate oxidative damage by regulating the expression of the glutathione peroxidase gene *MhGPX6* and enhancing the enzyme activities of POD and CAT under KCl stress.

In addition to the ion transporters and antioxidant enzyme genes, the expression levels of kinases (MhMAPK3, MhANP2, and MhGK) and transcription factors (MhERF017, MhMYB39, and MhWRKY28) screened out from RNA-Seq data under KCl stress were also detected. MAPK3 participates in the signaling pathway of salt stress in *Arabidopsis*, soybean, cucumber, and other plants (Im et al., 2012; Liu J. et al., 2020). *ANP2* is a gene of the MAPKKK family associated with NPK1 and is an important kinase in abiotic stress in rice (Ning et al., 2008; Lian et al., 2018). G-protein kinase plays an active role in plant response to salt stress (Shen et al., 2019). In this study, the expression of *MhMAPK3*, *MhANP2*, and *MhGK* was induced by KCl stress but inhibited by Res treatment (Figure 6). These results indicated that these three kinase genes would take part in responding to KCl and Res treatment. ERF, MYB, and WRKY transcription factors serve as connecting links between the upstream signal and the expression of functional genes under salt stress (Wang et al., 2019). MYB46 remarkably improves the salt tolerance of *Betula platyphylla* (Guo R. et al., 2017), and MdWRKY28 is an important regulator in apple salt adaptation (Dong et al., 2020). The data showed that the expression levels of *MhMYB39* and *MhWRKY28* were induced by KCl stress and exogenous Res treatment, indicating their important role in KCl and Res signaling transduction pathway. Furthermore, the variation tendency of *MhERF017* was similar to that of the three kinase genes (*MhMAPK3*, *MhANP2*, and *MhGK*), Na^+^/K^+^ transporter genes (*MhHAK5*, *MhKAT1*, *MhTPK1*, *MhNHX1*, and *MhNHX2*), and glutathione peroxidase gene *MhGPX6*, indicating their potential relationship. These kinases, transcription factors, ion transporters, and Res-signaling genes might have complicated regulation and interaction mechanisms. Future research will focus on this relationship and the Res-signaling transduction pathway under abiotic stress.

## Data Availability Statement

The original contributions presented in the study are included in the article/Supplementary Material, further inquiries can be directed to the corresponding author/s.

## Author Contributions

XZ and CW planned and designed the research. TL, YL, XX, ZS, CM, GS, and YT performed the experiments, conducted the fieldwork, and analyzed the data. XZ and TL wrote the manuscript. All authors contributed to the article and approved the submitted version.

## Conflict of Interest

The authors declare that the research was conducted in the absence of any commercial or financial relationships that could be construed as a potential conflict of interest.

## Abbreviations

Res: resveratrol
ROS: reactive oxygen species
MDA: malondialdehyde
SOD: superoxide dismutase
POD: peroxidase
CAT: catalase
ABA: abscisic acid
JA: jasmonate
CTK: cytokinin.
