# Supplementary material for: Resveratrol Alleviates the KCl Salinity Stress of Malus hupehensis Rhed
Source: Front Plant Sci. 2021 May 12;12:650485. doi: 10.3389/fpls.2021.650485 (PMC8149799; doi:10.3389/fpls.2021.650485)
Supplement: Supplementary file 1 [file Data_Sheet_1.PDF]

Supporting information

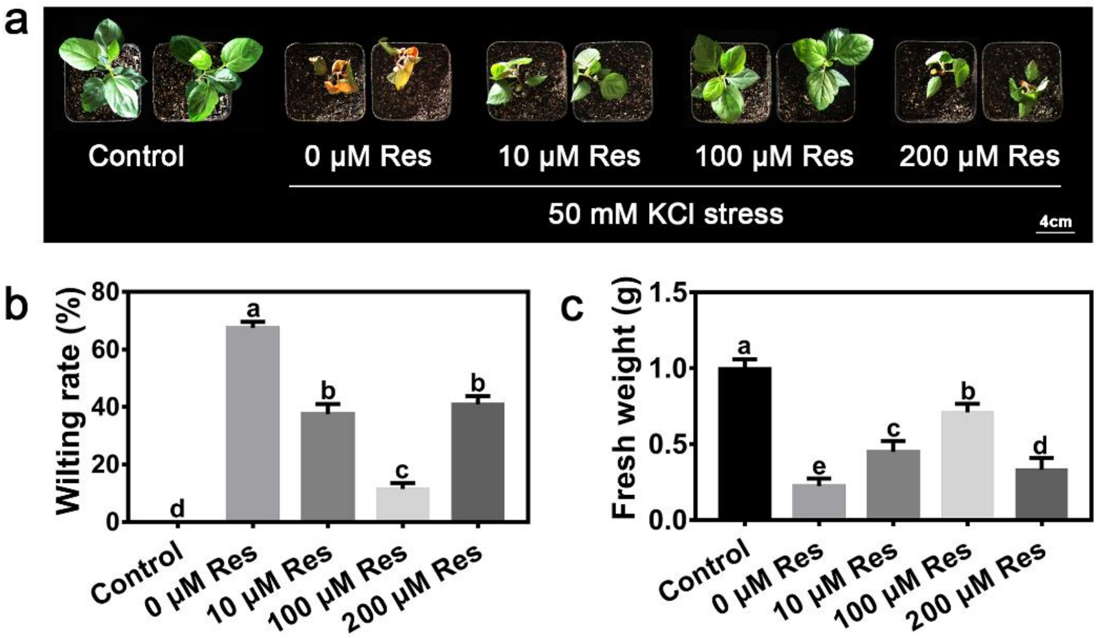

Figure S1 Phenotypes of *M. hupehensis* seedlings treated with 50 mM KCl stress and exogenous different concentrations of Res (0 μM, 10 μM, 100 μM, and 200 μM) after KCl stress for 15 days. Effect of different concentrations of Res on wilting rate (b) and fresh weight (c) of apple seedlings after KCl stress for 15 days. The data represent the mean ± SD of biological replicates. Different lowercase letters indicate significant differences, according to Fisher's LSD (P <0.05).

Table S1 The primers used for qRT-PCR

Primer name	Forward primer	Reverse primer
qMhSKOR	CATCCTGACAACTGGTGGTATCG	AAGTACCTCAGAGCAATCCGTTT
qMhHAK5	TTAATTTGAGTGTGTGAGAAGTGGCT	CTGTGAAATGGCGTGACTCG
qMhKAT1	CTTTACCTCTCCTTTTGTTCCCTAC	GCAACAGTGTCCATTTGGTATTC
qMhTPK1	GTCCACCGCAAATCCAGACTA	GAAGAGGCATGTCGTCGTAAAC
qMhNHX1	TTCTGCGTGAACTTTAGACCCT	AAGACTGAGATTTCCTTTCAAGC
qMhNHX2	CCACATTGATTCCAGTATTGCTT	CTCTTGAACTCTCCGTCACATTG
qMhCAX5	AATGTAGACCAAATAACAGGGAGG	CTGCTCTTGTTGTCGTCGTTGT
qMhCHX15	ATGGTTCAATAGTATGTTACGCTCC	GAATTTATCACTCTTTCCAAGCACC
qMhSOS1	TACACTGTCGCTCTGCTCATCC	CCAGTCGTAAGGGAAAGTGAGC
qMhGPX6	TTCCGAGAGTAAATCAATCCACG	AGGCAAACTCTACAATCTCGTCA
qMhPER65	GGCATTCTATTCCCATTCCCTT	GAGTTGGAAGCGATGAGGAGG
qMhpoxN1	GCTCCTCCAAATCATTGTTACTG	AAGAAGGACAGAAGCATCACAAC
qMhERF017	ATGTCAAACCACTTCTCCAAAATCT	GGAAATTAAACTTGGCGGTGC
qMhMYB39	AATGGGAATTGATCCTGTCACC	TGAGGTTGGATTTGGGGGTT
qMhWRKY28	ATCAGGAACCGAAAGACCTTTACTA	ATCGCCTTCAATAGACGAAAATAGT
qMhMAPK3	GCTACATGGGACATGACATTACTA	CCAAGATGTTCCTGCCTTTTAT
qMhANP2	AGCATTTAGCCATTCAGGCATAC	GCAAAAAAGACGAGCTGGAGAG
qMhGK	ACTCTGTTTGTGAAGGTTCGGTCTA	ACAAGATTACTGAGCTTTCCAGTGT
ACTIN1	CTTCAATGTGCCTGCCATGTAT	AATTTCCCGTTCAGCAGTAGTG
